# Supplementary material for: Evidence of exposure to SARS-CoV-2 in cats and dogs from households in Italy
Source: Nat Commun. 2020 Dec 4;11:6231. doi: 10.1038/s41467-020-20097-0 (PMC7718263; doi:10.1038/s41467-020-20097-0)
Supplement: Supplementary file 1 — Supplementary Information [file 41467_2020_20097_MOESM1_ESM.pdf]

Supplementary Table 1. Oligonucleotides used in the real-time RT-PCR assays for SARS-CoV-2

Target gene	Primer/probe	Sequence (5'-3')
E gene	E_Sarbeco_F	ACAGGTACGTTAATAGTTAATAGCGT
	E_Sarbeco_P1	FAM-ACACTAGCCATCCTTACTGCGCTTCG-BHQ
	E_Sarbeco_R	ATATTGCAGCAGTACGCACACA
N gene	N_Sarbeco_F	CACATTGGCACCCGCAATC
	N_Sarbeco_P	FAM-ACTTCCTCAAGGAACAACATTGCCA-BHQ
	N_Sarbeco_R	GAGGAACGAGAAGAGGCTTG

FAM: 6-carboxyfluorescein; BBQ: black hole quencher.
